# Bimodal expression of *RHOH* during myelomonocytic differentiation: Implications for the expansion of AML differentiation therapy

**DOI:** 10.1002/jha2.128

**Published:** 2021-01-20

**Authors:** Sylvie Galiègue‐Zouitina, Qiangwei Fu, Céline Carton‐Latreche, Nicolas Poret, Meyling Cheok, Frédéric Leprêtre, Martin Figeac, Bruno Quesnel, Hassiba El Bouazzati, Carl S Shelley

**Affiliations:** ^1^ JPARC UMRS 1172 Inserm Lille University Lille France; ^2^ Place de Verdun Institut pour la Recherche sur le Cancer de Lille Lille Cedex France; ^3^ California Institute for Biomedical Research La Jolla California USA; ^4^ Canther UMR 1277 Inserm‐9020 CNRS Lille University Lille France; ^5^ UMS 2014 ‐ US 41 Plateau de Génomique Fonctionnelle et Structurale Lille University Lille France; ^6^ CHU Lille Service des Maladies du Sang Lille France; ^7^ LLC Leukaemia Therapeutics Hull Massachusetts USA

**Keywords:** AML, differentiation therapy, myeloid differentiation, *RHOH*

## Abstract

RhoH is an unusual member of the Rho family of small GTP‐binding proteins in that it lacks GTPase activity. Since the RhoH protein is constantly bound by GTP, it is constitutively active and controlled predominantly by changes in quantitative expression. Abnormal levels of *RHOH* gene transcripts have been linked to a range of malignancies including acute myeloid leukemia (AML). One of the hallmarks of AML is a block in the normal program of myeloid differentiation. Here we investigate how myeloid differentiation is controlled by the quantitative expression of *RHOH*. Our analysis demonstrates that increasingly mature myeloid cells express progressively lower levels of *RHOH*. However, as monocytic myeloid cells terminally differentiate into macrophages, *RHOH* expression is up‐regulated. This up‐regulation is not apparent in AML where myeloid differentiation is blocked at stages of low *RHOH* expression. Nevertheless, when the up‐regulation of *RHOH* is forced, then terminal macrophage differentiation is induced and the Cdc42 and Wnt intracellular signalling pathways are repressed. These results indicate that *RHOH* induction is a driver of terminal differentiation and might represent a means of effecting AML differentiation therapy. The potential of this therapeutic strategy is supported by forced up‐regulation of *RHOH* reducing the ability of AML cells to produce tumours *in vivo*.

## 1 INTRODUCTION

RhoH is a member of the atypical family of Rho GTP‐binding proteins that also includes Rnd1‐3, RhoBTB1‐2, RhoU, and RhoV [1]. In common with classic Rho proteins, atypical members also need to bind GTP to be functional. However, in contrast to classic members, atypical members either lack the ability to hydrolyze GTP or exhibit a high intrinsic guanine nucleotide exchange rate. Consequently, atypical members are active constitutively and are regulated by post‐translational modifications, subcellular localization, and quantitative levels of expression [1].

In non‐malignant cells RhoH protein expression appears confined to hematopoietic cells. Here its subcellular localization, state of phosphorylation, and quantitative levels regulate differentiation, activation, intercellular interactions, proliferation, survival, homing, and chemotaxis [2, 3, 4, 5, 6, 7, 8, 9, 10, 11, 12, 13]. High levels of RhoH protein keep leukocytes quiescent by antagonizing the function of Rac1, RhoA, and Cdc42 through inhibiting their association with the plasma membrane [6, 8]. However, during leukocyte activation, RhoH protein redistributes away from Rac1, RhoA, and Cdc42. In addition, the quantitative levels of RhoH are reduced by a combination of lysosomal degradation and transcriptional repression of the *RHOH* gene by JunD [7, 14, 15]. This redistribution and quantitative down‐regulation relieves Rho signalling from repression by RhoH and consequently induces leukocyte activation, differentiation, adhesion, migration, proliferation and protection against apoptosis [2, 4, 6, 8‐10, 12]. In addition to its ability to control intracellular signalling by repression, RhoH protein can also positively affect T‐lymphocyte activation by recruiting the tyrosine kinases Zap70 and Lck to the T‐cell receptor and transporting the transcription factor kaiso to the nucleus [16, 17, 18, 19]. RhoH protein also binds Syk to positively affect mast cell activation mediated by FcεRI [20].

In malignant disease, aberrant somatic hypermutation and rearrangement of the *RHOH* gene have been found associated with a range of lymphomas [21, 22, 23, 24, 25, 26, 27, 28, 29, 30, 31, 32, 33, 34, 35, 36]. In addition, abnormal quantitative levels of *RHOH* expression have been linked to the pathogenesis of hairy cell leukemia, acute myeloid leukemia, chronic lymphocytic leukemia, and prostate cancer [37, 38, 39, 40]. In the case of hairy cell leukemia, reconstitution of appropriate *RHOH* expression protects against disease progression and mortality in a xenograft mouse model [37]. *RHOH* mRNA also acts as a biomarker for metastatic melanoma [41] and identifies brain, breast, colorectal, hepatobiliary, lung, and pancreatic cancer in platelet biopsies [42]. Little is known about the function of *RHOH* in myeloid cells. Therefore, we sought to address this void.

## 2 METHODS

### 2.1 Ethics statement

Informed consent for all human studies was obtained in accordance with the Declaration of Helsinki and approved by the ethics committee of Saint‐Germain‐en‐Laye, Paris, France, the internal review board of the Centre Hospitalier Universitaire, Centre de Biologie, Lille, France or the institutional review board of the French Regulatory Agency. All animal protocols were approved by the Institutional Animal Care and Use Committee of the University of Wisconsin, La Crosse, Wisconsin, USA.

### 2.2 Human blood samples

Transcriptome analyses were performed on cryopreserved peripheral blood mononuclear cells obtained between 2008 and 2010 from patients diagnosed with FAB subtyped *de novo* AML by the ALFA‐0701 study of the Acute Leukaemia French Association [43, 44, 45]. Staining with May Grünwald Giemsa demonstrated that 70 ‐ 90% of these cells were AML blasts. Western blot analyses of protein expression were performed on cells originating from the peripheral blood of healthy volunteers and patients diagnosed at the Service des Maladies du Sang, Lille, France with FAB subtyped *de novo* AML. Mononuclear cells were prepared from these peripheral blood draws by Ficoll gradient centrifugation then AML blasts or normal monocytes were isolated by negative selection using, respectively, the EasySep™ APC Selection Kit II (STEMCELL Technologies, Inc., Grenoble, France) or the Monocyte Isolation Kit II (Miltenyi Biotec, SAS, Paris, France). Staining with May Grünwald Giemsa demonstrated that the cells isolated from patients were 70 ‐ 97% AML blasts and cytometry showing the cell surface presence of CD14 but absence of CD3 and CD19 demonstrated that 90% of the cells isolated from healthy volunteers were monocytes.

### 2.3 Cell culture

The AML cell lines KG‐1, KG‐1a, Kasumi‐3, OCI‐AML3, MV4‐11, and THP‐1 were obtained from the American Type Culture Collection (ATCC) (Manassas, Virginia USA). The AML cell lines SKM‐1, SHI‐1, YNH‐1, GF‐D8, and HL60 were obtained from the Deutsche Sammlung von Mikroorganismen and Zellkulturen GmbH (DSMZ) (Braunschweig, Germany). The AML cell lines Nomo‐1, Mono Mac 1 and Mono Mac 6 were obtained from Dr. Meyling Cheok (Canther, UMR 1277 Inserm‐9020 CNRS, Lille) and the AML cell line Molm‐14 was kindly provided by Dr. Brian J. Druker (Knight Cancer Research Institute, Oregon Health & Science University, Portland, Oregon, USA). The cell lines KG‐1, YNH‐1, HL‐60, Nomo‐1, Mono Mac 1, Mono Mac 6, MV4‐11, Molm‐14, and THP‐1 were grown in RPMI 1640 containing 10% heat‐inactivated (v/v) fetal bovine serum (FBS). The cell lines Kasumi‐3, GF‐D8, KG‐1a, and SKM‐1 were grown in RPMI 1640 containing 20% (v/v) heat‐inactivated FBS. The cell line SHI‐1 was grown in IMDM containing 15% heat‐inactivated (v/v) FBS. SKM‐1, MV4‐11, YNH‐1, and GF‐D8 cells were grown in the presence of 1, 5, 10, and 50 ng/ml GM‐CSF, respectively. Where indicated, HL60 and OCI‐AML3 were treated with 20 ng/ml PMA, 100 ng/ml LPS, or equal volumes of their vehicles DMSO and PBS, respectively. OCI‐Empty and OCI‐RhoH were grown in alpha‐MEM containing 20% (v/v) heat‐activated FBS and 150 μg/ml hygromycin B. All cell lines were cultured in the presence of 100 Units/ml penicillin and 100 μg/ml streptomycin. Authentication of cell lines was performed by STR profiling and certificates were obtained from the ATCC Cell Authentication Testing Service. AML patient blasts and normal monocytes were cultured in RPMI 1640 containing 20% heat‐inactivated (v/v) FBS and 4 mM L‐Glutamine in the presence of 5 ng/ml PMA or an equal volume of DMSO.

### 2.4 Plasmid construction and generation of stable cell line pools

The construction of the expression plasmids pMEP4‐Empty and pMEP‐RhoH has been described previously [37]. These plasmids were linearized by digestion with *Cla*I and then independently transfected by electroporation into the AML cell line OCI‐AML3. Pools of OCI‐AML3 cells that contained the transfected plasmids stably integrated within their genome were selected by treatment with 150 μg/ml of hygromycin B. The cell line pool stably expressing pMEP‐Empty was named OCI‐Empty and the pool expressing pMEP‐RhoH was named OCI‐RhoH.

### 2.5 Transcriptome datasets of FAB subtyped AML

*RHOH* expression was analyzed in the transcriptomes of FAB subtyped *de novo* AML acquired by five microarray studies and one RNA‐seq study. The microarray datasets were from the multi‐center ALFA‐0701 study [43, 44, 45], the Gene Expression Omnibus identifiers GDS1064 [46], and GDS3057 [47] for AML samples with all karyotypes and GDS3312 and GDS3329 for AML samples with a normal karyotype [48]. All these microarray datasets were produced using Human Genome U133 Plus 2.0 Affymetrix arrays containing the *RHOH* Affymetrix probe 204951_at and signal normalization either by robust multi‐array averaging or application of a variance stabilizing algorithm. The RNA‐seq datasets analyzed for *RHOH* expression spanned The Cancer Genome Atlas identifiers TCGA.AB.2802 ‐ TCGA.AB.3012 but excluded the identifiers where FAB subtype was unassigned or assigned M3, M6 or M7 [45, 49]. These expression data were normalized by Expectation Maximization [50].

### 2.6 Quantitative RT‐PCR analyses

Templates of cDNA were prepared from total RNA as described previously [14, 37, 51]. Quality and quantity of templates were evaluated by analysis of *ABL* mRNA, as recommended by Europe Against Cancer [52]. The B‐lymphocyte cell line Raji that expresses high levels of both *RHOH* and *ABL* was used to produce calibration curves that evaluated PCR efficiency. Evaluation of the sum total of all *RHOH* gene transcripts was achieved using the same primers and the same *RHOH* TaqMan probe as previously described [37]. The relative level of endogenous *RHOH* expression was calculated by dividing the quantitative level of *RHOH* expression by the quantitative level of *ABL* expression, again as previously described [37].

### 2.7 Western blotting

Proteins were isolated from cells using M‐PER™ lysis reagent or RIPA buffer (Fisher Scientific France SAS, Illkirch, France). Proteins were then reduced, subjected to polyacrylamide gel electrophoresis, and transferred to nitrocellulose filters. Next, filters were incubated with primary antibodies directed against human RhoH, glyceraldehyde 3‐phosphate dehydrogenase (GAPDH), heat shock protein 70 (HSP70), JunD, CD11b, or CD93. Analysis of RhoH protein expression utilized the rabbit polyclonal antibody RhoH‐V generated against the sequence CVNAMEGKKLAQDVRAK representing amino acids 130 to 146 of the human RhoH protein (Covalab SAS, Villeurbanne, France) [53]. Western blot analysis of GAPDH, HSP70, and JunD was performed using, respectively, the rabbit polyclonal antibody FL‐335 and the mouse monoclonal antibodies B‐9 and D‐9 purchased from Santa Cruz Biotechnology, Inc. (Heidelberg, Germany). Western blot analysis of CD11b and CD93 expression was performed using, respectively, the rabbit polyclonal antibodies ab134690 and ab231945 purchased from Abcam plc, (Cambridge, UK). Following incubation with primary antibodies, filters were washed and incubated with appropriate anti‐mouse or anti‐rabbit secondary antibodies conjugated with horseradish peroxidase (Cell Signalling Technology Europe, B.V., Leiden, The Netherlands). Filters were again washed and peroxidase visualized using the Amersham ECL Prime Western Blotting System and quantitated using an ImageQuant™ LAS 4000 instrument equipped with Image J software (GE Healthcare Europe, GmbH, Freiburg, Germany).

### 2.8 Flow cytometry

Phenotypic validation of monocytes isolated from healthy donors was performed by flow cytometry analysis using the PE‐conjugated anti‐CD14 mouse monoclonal antibody TÜK4 (Miltenyi Biotec, SAS, Paris, France), the FITC‐conjugated anti‐CD3 mouse monoclonal antibody UCHT1 (Becton Dickinson France, SAS, Le Pont de Claix, France) and the PE‐conjugated anti‐CD19 mouse monoclonal antibody J3.119 (Beckman Coulter France, SAS, Villepinte, France). All FACS analyses were performed using a Cyan ADP flow cytometer equipped with Summit 4.3 software (Beckman Coulter France, SAS, Villepinte, France).

### 2.9 Differential microarray analysis

Total RNA was isolated from the daughter cell lines OCI‐RhoH and OCI‐Empty using the RNAeasy kit from Qiagen that included a DNAse I treatment. Control of RNA quality was evaluated using an Agilent 2100 Bioanalyser (Agilent Technologies France, SAS, Les Ulis, France). Total RNA was then processed and analyzed on Agilent‐026652 Whole Human Genome Agilent 4 × 44K v2 microarrays using the kits for cRNA amplification, labeling, fragmentation, hybridization, and washing recommended by the manufacturer for the Two‐Color Microarray‐Based Gene Expression protocol (Agilent Technologies France, SAS, Les Ulis, France). For each cell line, two independent biological samples were studied by dye‐swap analysis. Microarrays were scanned using an Agilent DNA Microarray G2505B scanner and the expression data quantitated by Agilent Feature Extraction Software© (FE version 10.7.3.1) (Agilent Technologies France, SAS, Les Ulis, France). This same software was used for background subtraction, LOWESS normalization and statistical analysis. Comparison of the transcriptome of OCI‐RhoH with that of OCI‐Empty demonstrated a significant difference in the expression of 72 genes as defined by a *P* value of 0.05 or less calculated using the Student's *t‐*test. These data are publically available at the Gene Expression Omnibus repository under accession number GSE138479.

### 2.10 Xenograft mouse model

Female mice that were 4 weeks old and of the strain SHrN^TM^ Hairless NOD.SCID were purchased from Harlan Laboratories, Inc. (Indianapolis, IN, USA). Mice were housed in sterilized Super Mouse 750 Micro‐Isolator ventilated cages on a RAIR Isosytem rack (Lab Products, Inc., Seaford, DE, USA). This housing system was kept in a barrier room accredited by AAALAC‐I that was designed for the husbandry and surgery of immunodeficient animals. Mice were monitored daily and sterile water and Teklad Global 18% Protein Rodent Diet (Harlan Laboratories, Inc., Indianapolis, IN, USA) were provided *ad libitum*. Mice were provided with sterile Teklad Sani‐Chips for bedding and iso‐BLOX nesting material (Harlan Laboratories, Inc., Indianapolis, IN, USA) as well as LifeSpan Rodent Enrichment (Lab Products, Inc., Seaford, DE, USA). Social interaction was ensured by housing mice in groups of two to three per sterile cage. Mice were anesthetized by initial inhalation of 2.5% then subsequent inhalation of 2% isoflurane delivered by facemask using a SurgiVet Universal CDS 9000 Small Animal Anesthesia Machine (Smiths Medical, Dublin, OH, USA). During maintenance anesthesia, mice were taped dorsal‐side up to a Homeothermic Blanket System set at 37°C (DC Temperature Control System, FHC, Inc., Bowdoin, ME, USA). Ophthalmic ointment was applied to the eyes to prevent corneal drying and trauma. The dorsal right or left hind flank was swabbed with 70% ethanol and here 5 or 10 × 10^6^ of the cell line OCI‐Empty or its sister OCI‐RhoH in a volume of 200 μl were injected subcutaneously using a 27‐gauge needle with the bevel facing up. Injected cells originated from exponentially growing cultures that had been washed twice in sterile PBS. Injections were performed at a 45‐degree angle and aspiration monitored the absence of blood vessel entry. After removing the needle, the injection site was held firmly with sterile gauze for approximately 30 seconds. Buprenorphine at a concentration of 0.1 mg kg^−1^ was injected subcutaneously and then mice were returned to their cages to recover from anaesthesia. Three weeks after injection, all mice were killed by cervical dislocation following anaesthesia effected by intraperitoneal injection of a mixture of 160 mg kg^−1^ ketamine plus 10 mg kg^−1^ xylazine. At this point, tumours were dissected free of the skin and body tissue and their maximum length measured in three dimensions with electronic callipers. Using these dimensions, tumour volume was calculated as an ellipsoid according to the formula: (4 x π x L x W x T) / 3, where L is the length, W is the width and T is the thickness. All animal use protocols were approved by the Institutional Animal Care and Use Committee of the University of Wisconsin, La Crosse, Wisconsin, USA.

## 3 RESULTS

### 3.1 *RHOH* expression progressively declines during the early and intermediate stages of myeloid differentiation

**FIGURE 1:**
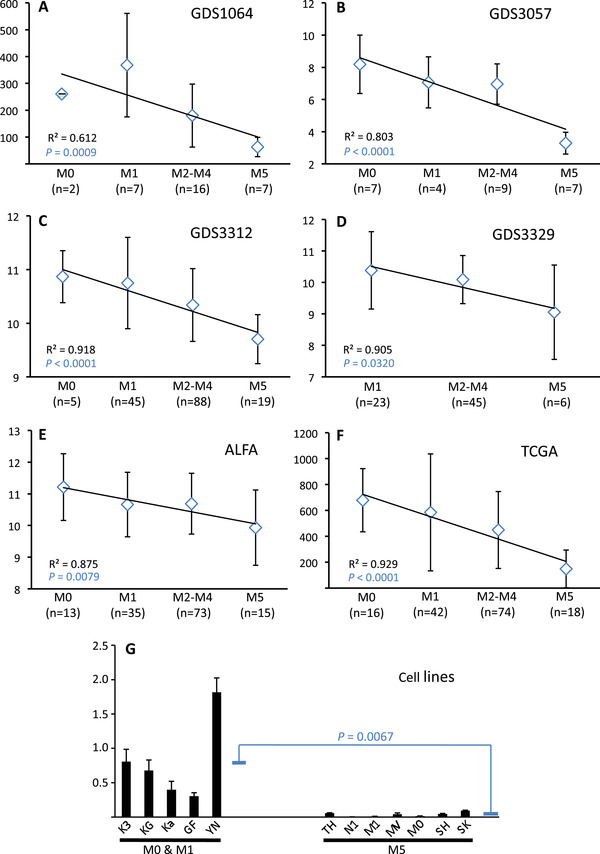
Repression of *RHOH* mRNA expression during the intermediate phases of myelomonocytic differentiation as assessed by the FAB subtype of AML patient samples and cell lines. (**A– D**) Regression curves of the means of relative *RHOH* mRNA levels as a function of FAB subtype established from the NCBI GEO Datasets GDS1064 [46] and GDS3057 [47] composed of AML patients with all karyotypes and GDS3312 and GDS3329 composed only of AML patients with a normal karyotype [48]. (**E**) Regression curves of the means of relative *RHOH* mRNA levels as a function of FAB subtype established from microarray analyses of AML patients recruited by the ALFA‐0701 study (ALFA) [43, 44, 45]. (**F**) Regression curve of the means of relative *RHOH* mRNA levels as a function of FAB subtype established from normalized RNA‐seq data generated by The Cancer Genome Atlas (TCGA) [45, 49]. The GDS DataSets as well as the ALFA‐0701 and TCGA data all reveal a linear decrease in *RHOH* mRNA expression as differentiation stage progresses from AML FAB subtype M0 to M5 (R^2^ values are presented at the bottom left of each panel in black text). In addition, all sets of data demonstrate a significant difference in *RHOH* mRNA expression between the early M0 and M1 stages of AML differentiation compared to the later M5 stage (*P* values presented at the bottom left of each panel in blue text). Mean *RHOH* mRNA levels for individual FAB subtypes are depicted as open blue lozenges with their standard deviations (SD) presented as vertical bars above and below. In addition, the number (n) of patients analysed in each FAB subtype is presented. (**G**) Total RNA was extracted from the cell lines Kasumi‐3 (K3), KG‐1 (KG) and KG‐1a (Ka) established from patients diagnosed with AML FAB subtype M0; from the cell lines GF‐D8 (GF) and YNH‐1 (YN) representing AML FAB subtype M1; and from THP‐1 (TH), Nomo‐1 (N1), Mono Mac‐1 (M1), MV4‐11 (MV), Molm‐14 (MO), SHI‐1 (SH) and SKM‐1 (SK) representing FAB subtype M5. The relative level of *RHOH* mRNA expression was evaluated for each cell line by Q‐RT‐PCR as previously described [37]. The mean relative expression of *RHOH* mRNA for three independent cultures of each cell line, each analysed twice in duplicate by Q‐RT‐PCR, are depicted as histograms with SD presented as vertical lines above. The mean relative *RHOH* mRNA expression in FAB subtypes M0 and M1 combined is 0.802 ± SD 0.603 while that of M5 is 0.037 ± SD 0.033. These two means are depicted as blue horizontal bars and their difference of 21.7 fold was determined to be significant (*P* = 0.0067). The *P* values presented in all panels were calculated using an unpaired Student's *t*‐test

**FIGURE 2:**
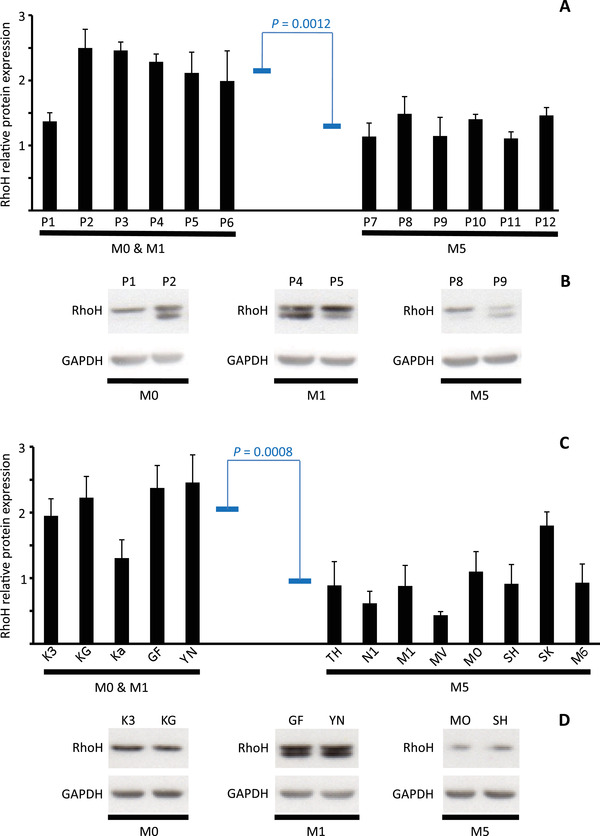
Repression of RhoH protein expression during the intermediate phases of myelomonocytic differentiation as assessed by the FAB subtype of AML patient samples and cell lines. (**A**) Western blot evaluation of relative RhoH protein expression in lysates prepared from peripheral blood mononuclear cells of patients diagnosed with AML FAB subtype M0 (P1 and P2), M1 (P3 ‐ 6) or M5 (P7 ‐ 12). RhoH protein expression is normalized to the expression of GAPDH protein and presented as histograms representing the mean ± SD of three (P1, P2, P5, P6, and P8 ‐ 12) or four (P3, P4, and P7) independent Western blot analyses. Blue horizontal bars represent the means of relative RhoH protein expression in FAB subtypes M0 and M1 (2.12 ± SD 0.42) compared to FAB subtype M5 (1.29 ± SD 0.18). The difference between these two means was calculated by an unpaired Student's *t*‐test as being significant (*P* = 0.0012). (**B**) Representative Western blots evaluating RhoH and GAPDH protein expression in patients with AML FAB subtype M0 (P1 and P2), M1 (P4 and P5), and M5 (P8 and P9). (**C**) Western blot evaluation of relative RhoH protein expression in lysates prepared from cell lines Kasumi‐3 (K3), KG‐1 (KG), and KG‐1a (Ka) representing AML FAB subtype M0, cell lines GF‐D8 (GF) and YNH‐1 (YN) representing FAB subtype M1 and THP‐1 (TH), Nomo‐1 (N1), Mono Mac‐1 (M1), MV4‐11 (MV), Molm‐14 (MO), SHI‐1 (SH), SKM‐1 (SK), and Mono Mac‐6 (M6) representing FAB subtype M5. RhoH protein expression is normalized to the expression of GAPDH protein and presented as histograms representing the mean ± SD of the results obtained from two or three independent cultures of each cell line and each of these subjected to between two and eight Western blot analyses. Blue horizontal bars represent the means of relative RhoH protein expression in FAB subtypes M0 and M1 (2.06 ± SD 0.46) compared to FAB subtype M5 (0.94 ± SD 0.40). The difference between these two means was calculated by an unpaired Student's *t*‐test as being significant (*P* = 0.0008). (**D**) Representative Western blots evaluating RhoH and GAPDH protein expression in cell lines established from patients with AML FAB subtype M0 (K3 and KG), M1 (GF and YN), and M5 (MO and SH)

**FIGURE 3:**
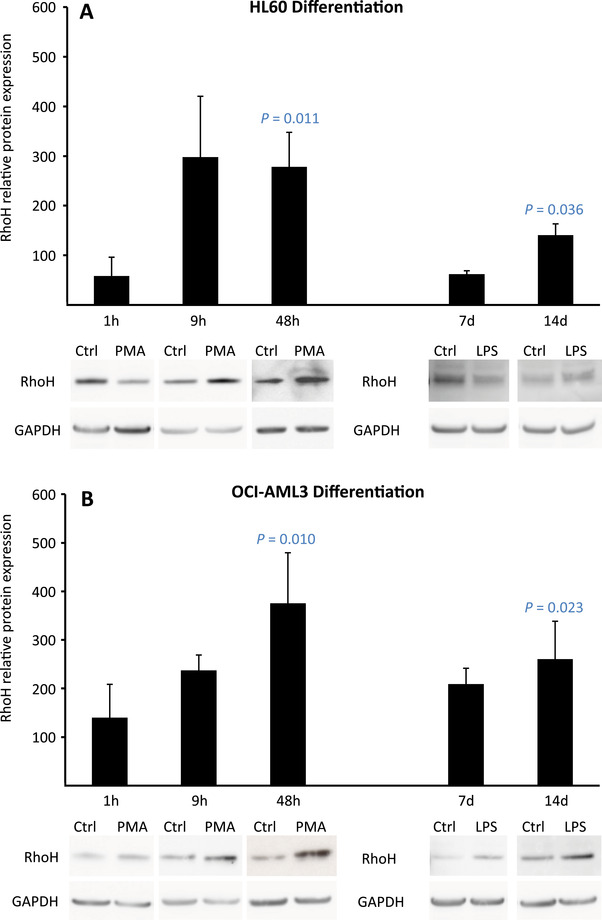
Induction of RhoH protein expression during terminal differentiation of AML cell lines. (**A**) The AML cell line HL60 or (**B**) the AML cell line OCI‐AML3 were cultured in the presence of 20 ng/ml PMA or its vehicle DMSO (Ctrl) for 1, 9 or 48 h or 100 ng/ml LPS or its vehicle PBS (Ctrl) for 7 or 14 days. Total protein lysates were prepared and Western blot analysis of RhoH and GAPDH protein expression performed. Histograms represent the means ± SD of RhoH protein expression normalized to GAPDH protein in cells treated with PMA or LPS calculated relative to values of 100% assigned to normalized levels of RhoH protein expressed in cells treated with the appropriate vehicle. Protein lysates were prepared from three independent cell cultures for each treatment condition. Each lysate was analyzed for RhoH and GAPDH expression one or two times by Western blotting. One of these analyses is presented below each histogram. *P* values indicating significant induction of RhoH protein expression in cells treated with PMA for 48 hours or LPS for 14 days compared to cells treated with the appropriate vehicle were calculated using an unpaired Student's *t*‐test

Interrogation of four independent data sets in the Genome Expression Omnibus indicated that AML subtypes originating from increasingly mature myeloid cells exhibited progressively lower levels of *RHOH* mRNA expression (Figure 1A‐D) [46, 47, 48]. Analysis of the AML data set produced by The Cancer Genome Atlas and a data set produced by the multicenter ALFA‐0701 study demonstrated this same repression profile (Figure 1E‐F) [43‐45, 49]. In addition, cell lines derived from M5 AML patients showed lower *RHOH* mRNA expression than cell lines derived from M0 and M1 AML patients (Figure 1G). Since the quantitative level of mRNA is not necessarily a direct reflection of the level of expression of its translation product, we analyzed by Western blotting RhoH protein expression. This analysis indicated that, consistent with *RHOH* mRNA, RhoH protein was lower in myeloid cells of AML patients with subtype M5 compared to the less mature malignant cells of M0 and M1 patients (Figure 2A, B). Additional analysis demonstrated that cell lines representing more mature myeloid cells also express lower levels of RhoH protein compared to lines derived from earlier stages of differentiation (Figure 2C, D). Furthermore, when the AML cell line HL60 was induced to differentiate, with either phorbol ester for 1 hour or lipopolysaccharide for 7 days, RhoH protein levels were again repressed (Figure 3A). Taken together, analysis of AML patients and the HL60 cell line indicates that progressive *RHOH* repression is a dynamic indicator of early to middle stage myeloid differentiation.

### 3.2 *RHOH* expression is induced during the terminal stages of myeloid differentiation

**FIGURE 4:**
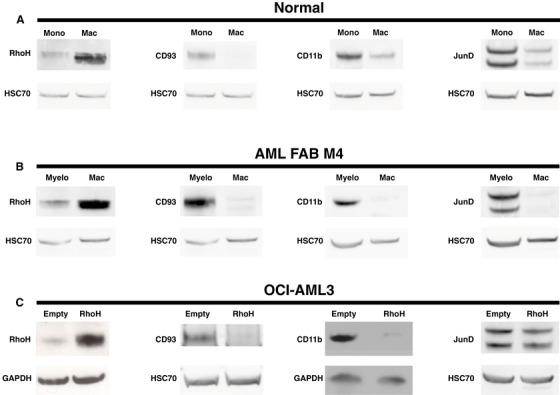
Induction of RhoH protein expression during terminal differentiation of freshly isolated monocytes and myeloblasts; RhoH induction drives terminal differentiation (**A**) Normal monocytes were isolated from the peripheral blood of healthy donors. Total protein lysates were prepared from these cells (Mono). In addition, total protein lysates were prepared after these monocytes were induced to differentiate into macrophages with 5 ng/ml of PMA for 48 hours (Mac). Next, Western blot analysis was performed to determine the expression of RhoH, CD93, CD11b, and JunD proteins relative to the control protein HSC70. Depicted are representative examples of analyses performed on five independent preparations of monocytes and macrophages. Three preparations were analysed for RhoH and CD93 expression by four and two Western blots, respectively. Two preparations were analysed for CD11b and JunD expression by one and two Western blots, respectively. (**B**) Myeloblasts were isolated at diagnosis from patients with AML FAB M4. Total protein lysates were prepared from these cells (Myelo). In addition, total protein lysates were prepared after these myeloblasts were induced to differentiate into macrophages with 5 ng/ml of PMA for 48 hours (Mac). Western blot analysis was performed to determine the expression of RhoH, CD93, CD11b and JunD proteins relative to the control protein HSC70. Depicted are representative examples of analyses performed on two independent preparations of myeloblasts and macrophages. Each preparation was analysed by two Western blots for RhoH and CD93 expression and by one Western blot for CD11b and JunD expression. (**C**) Total protein lysates were prepared from the AML cell line OCI‐AML3 stably transfected either with the empty vector pMEP4 (Empty) or pMEP4‐RhoH that constitutively expresses *RHOH* (RhoH). Next, Western blot analysis was performed to determine the expression of RhoH, CD93, CD11b and JunD proteins relative to the control proteins GAPDH or HSC70. Depicted are representative examples of analyses performed on three independent cultures of each stable transfectant. Each independent culture was analysed by three Western blots for RhoH expression and two Western blots for CD93, CD11b and JunD expression

In order to begin to determine the pattern of *RHOH* expression during terminal phases of myeloid differentiation, Western blot analysis was performed on HL60 and OCI‐AML3 cells induced for 9 and 48 hours with phorbol ester or lipopolysaccharide for 14 days. In all cases, an induction of *RHOH* expression was observed (Figure 3). Cell lines cultured *in vitro* do not necessarily mimic the molecular biology of their equivalent cell‐type as it exists *in vivo*. Therefore, the results obtained from HL60 and OCI‐AML3 were verified by analysis of monocytes isolated directly from healthy volunteers and myeloblasts isolated directly from patients diagnosed with AML FAB subtype 4. These monocytes and myeloblasts were treated with phorbol ester for 48 h and terminal differentiation into macrophages confirmed by their acquisition of a strong adhesive phenotype, morphological spreading, and characteristic down‐regulation of CD11b and CD93 protein expression (Figure 4A, B, Supplemental Figure S1) [54, 55]. Further analysis demonstrated that this process of terminal differentiation is also characterized by the induction of RhoH protein expression (Figure 4A, B). Consistent with this induction, expression of JunD that represses *RHOH* gene transcription is down‐regulated (Figure 4A, B) [14]. This same reciprocal change in expression of *JUND* and *RHOH* is found when monocytes are induced to differentiate into mature macrophages and subsequently M1 polarized (GEO Series accession number GSE5099) [56]. Furthermore, when the monocytic cell line THP‐1 is differentiated into mature cells with functions of macrophages the induction of *RHOH* and the concomitant repression of *JUND* are also observed (GEO Series accession number GSE53691) [57].

### 3.3 Induction of *RHOH* expression in AML cells drives terminal differentiation

In order to determine whether *RHOH* induction is a passenger or a driver of terminal myeloid differentiation we produced two daughter lines from the AML cell line OCI‐AML3 that exhibits low endogenous expression of *RHOH*. One daughter stably expressed an empty plasmid vector and was named OCI‐Empty and the second daughter stably expressing *RHOH* in the same vector was named OCI‐RhoH. Western blot analysis confirmed that RhoH protein expression was induced in OCI‐RhoH compared to OCI‐Empty (Figure 4C). Further analysis demonstrated that OCI‐RhoH exhibits lower expression of the proteins CD93 and CD11b compared to OCI‐Empty (Figure 4C). The down‐regulation of CD11b and CD93 protein expression are individual hallmarks of the differentiation of monocytes into macrophages (Figure 4A, B) [54, 55]. Consequently, *RHOH* induction appears to be a driver of this form of terminal myeloid differentiation.

### 3.4 *RHOH* induction is associated with acquisition of a mature myeloid phenotype and alters expression of genes controlling Cdc42 and Wnt signalling

**FIGURE 5:**
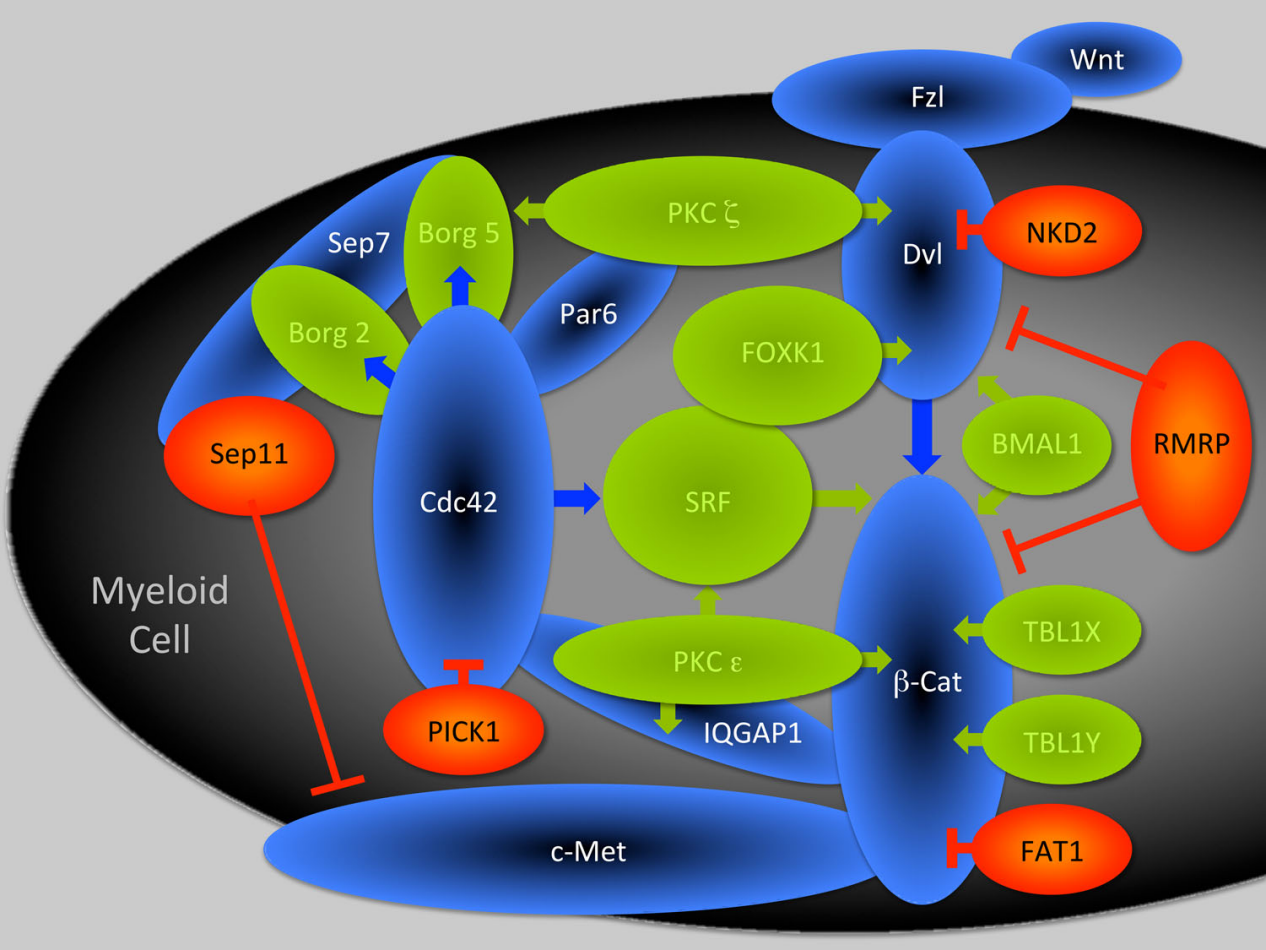
Regulators of Cdc42 and Wnt signalling controlled by *RHOH*. Differential microarray analysis identified 72 transcripts that are significantly changed in their expression when *RHOH* is reconstituted in the M4 AML cell line OCI‐AML3 (GEO Accession number GSE138479). Of these transcripts, 13 encode proteins and 1 the long non‐coding RNA *RMRP* known to regulate Cdc42 and/or Wnt signalling (Supplemental References 2). Core components of these signalling pathways are depicted as blue ovals. Agonists of these signalling pathways that are down‐regulated by *RHOH* induction are depicted as green ovals and antagonists up‐regulated by *RHOH* are depicted as red ovals. Overlapping ovals signify the direct binding of proteins

In order to gain insight into the molecular mechanisms by which the induction of *RHOH* might drive monocyte to macrophage differentiation, we compared the transcriptome of OCI‐RhoH with that of OCI‐Empty. This analysis identified 72 transcripts that were significantly altered in their expression upon *RHOH* induction (GEO Accession number GSE138479). Literature searches revealed that 42 of these 72 transcripts encode proteins or represent long non‐coding RNAs implicated in regulating functions characteristic of mature myeloid cells (Table 1). These functions appear to center on two main areas. The first is the control of processes essential to the journey of mature myeloid cells to their tissue sites of action such as adhesion, directional movement, and remodeling of the extracellular matrix. The second is the control of actions once arrived at tissue sites such as phagocytosis, the generation of reactive oxygen species (ROS), and apoptosis. Further analysis revealed that 9 of the transcripts down‐regulated by *RHOH* induction encode proteins that positively affect signalling through the Cdc42 and/or Wnt pathways while 4 transcripts up‐regulated by *RHOH* induction encode proteins that negatively affect Cdc42 or Wnt signalling. In addition, *RHOH* induction up‐regulates the long non‐coding RNA *RMRP* that is also known to repress Wnt signalling. Consequently, these results suggest that *RHOH* induction represses signalling through the interlinked Cdc42 and Wnt pathways by the coordinated down‐regulation of positive effectors and up‐regulation of negative effectors (Figure 5).

**TABLE 1:** Genes controlled by RHOH that regulate functions characteristic of mature myeloid cells

	Movement to tissue site					Action at tissue site		
	Cytoskeleton	Migration	Polarization	Adhesion	Matrix	Phagocytosis	ROS	Apoptosis
*ARNTL*	X	X	X	X	X	X	X	X
BARX1	X			X				
BMERB1	X							
*CD93*	X	X	X	X	X	X		
CDC42EP1	X	X	X	X				
CDC42EP3	X	X	X	X	X	X		
COL6A2	X	X		X			X	
COL15A1	X		X	X				
FOXK1	X						X	
KCNN4	X	X	X	X	X	X	X	
PLD3							X	
*PRAM1*						X		
*PRKCE*	X	X	X	X	X	X	X	X
*PRKCZ*	X	X	X	X	X	X	X	X
SLC4A2	X	X	X		X	X		X
SRF	X	X	X	X	X	X	X	X
TBL1X/Y	X			X				
TNN/TNW	X	X	X	X	X			
*TESC*		X			X	X	X	X
CCDC3		X		X				
*CDKN2C*				X			X	
*DUSP5P1*							X	
*FAT1*	X	X	X	X			X	X
GIMAP1							X	
GPR179		X		X				
HEPIS				X				
HSH2D							X	
IL17RC	X					X	X	
LHX1		X						X
LOC101927497	X							
MORC4							X	
*NKD2*		X	X		X			X
NPY	X	X	X	X	X	X	X	X
PEX7	X	X				X	X	
PICK1	X	X	X	X		X	X	X
POLR3G							X	
RMRP		X			X			X
*RPS3a*							X	
SEPTIN11	X					X		
*SNHG5*		X					X	X
SYNJ2BP	X			X	X			
TMEM132A			X				X	

Expression of genes listed in green text are down‐regulated when *RHOH* is induced. Genes listed in red text are up‐regulated when *RHOH* is induced. Abnormal regulation of genes listed in italic text has been linked to the pathogenesis of AML (Supplemental References 1).

### 3.5 Induction of *RHOH* expression limits tumour growth in a xenograft model of AML

**FIGURE 6:**
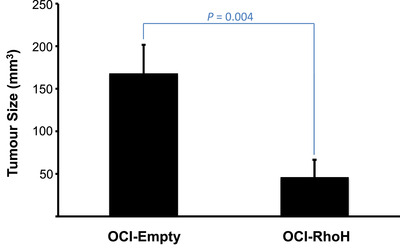
*RHOH* reconstitution inhibits the growth of AML tumours *in vivo*. The AML cell line OCI‐RhoH that stably expresses the *RHOH* vector pMEP‐RhoH and its sister line OCI‐Empty that stably expresses the same vector empty of *RHOH* were injected subcutaneously into SHrN^™^ Hairless NOD.SCID mice (Harlan Laboratories, Inc., Indianapolis, Indiana, USA). In one experiment 5 million OCI‐Empty cells were injected into 8 mice and in parallel 5 million OCI‐RhoH cells were injected into 7 mice. In a second experiment 10 million OCI‐Empty or OCI‐RhoH cells were each injected into 5 mice. The volumes of palpable tumours were measured 3 weeks after injection. Tumour volumes beyond 1.5 times the interquartile ranges of the ordered OCI‐Empty and OCI‐RhoH datasets were removed (Supplemental Table 1). Histograms represent the means ± SEM of the remaining volumes (OCI‐Empty n = 12; OCI‐RhoH n = 11). A Mann‐Whitney test determined that the mean volume of tumours produced by OCI‐RhoH was significantly smaller than that produced by OCI‐Empty (*P* = 0.004)

Transcriptome analysis implicates *RHOH* induction in regulating 42 genes controlling the function of mature myeloid cells. Strikingly, abnormal regulation of 12 of these genes has been functionally linked to the pathogenesis of AML (Table 1, italicized genes). In addition, *RHOH* induction is demonstrated to cause the down‐regulation of CD11b and CD93 protein expression characteristic of monocytes terminally differentiating into macrophages (Figure 4C) [54, 55]. Taken together, these considerations indicate that *RHOH* induction is a driver of terminal differentiation and might represent a means of AML treatment analogous to the differentiation therapy effected by all‐*trans* retinoic acid on the acute promyelocytic subtype [58]. In order to begin to determine the efficacy of potential *RHOH* differentiation therapy, we injected immunocompromised mice with the cell line OCI‐RhoH and assessed its ability to produce tumours compared to its sister line OCI‐Empty. In total 12 of 13 mice injected with OCI‐Empty developed tumours while only 5 of 12 mice injected with OCI‐RhoH did so. In total, the tumours produced by *RHOH* differentiated AML cells had average volumes 3.6 fold smaller than those produced by their non‐differentiated counterparts (Figure 6).

## 4 DISCUSSION

Iwasaki *et al* published in 2008 that low‐level expression of *RHOH* mRNA was an indicator of poor prognosis in AML [38]. They further reported that this low‐level expression is an unfavorable prognostic biomarker for both overall and disease‐free survival that is independent of age, white blood cell count, cytogenetics, and mutation of the *FLT3, NRAS, TP53*, and *NPM1* genes. These findings suggest that targeting abnormal *RHOH* expression might have therapeutic efficacy in a broad range of AML patients.

Apparently contradictory to our findings, Iwasaki *et al* found no significant differences in *RHOH* transcript levels between FAB subtypes. However, further scrutiny of their results in light of ours indicates we are in agreement. Iwasaki *et al* analyzed 1 patient classified as FAB M0, 22 as M1, 59 as either M2 or M4, 7 as M5, and 1 as M6. Where more than one patient was analyzed, low *RHOH* expression was exhibited by 8 out of 22 patients with the M1 subtype, 31 of 59 M2/M4 patients and 6 of 7 patients with subtype M5. Therefore, as myeloid differentiation reflected in FAB subtype progresses from M1 to M2/M4 to M5 the proportion of patients where *RHOH* expression is low steadily increases from 0.36 to 0.53 to 0.86, respectively. Consequently, viewed in this way, the study by Iwasaki *et al* confirms our observation that *RHOH* mRNA levels progressively decrease during the early and intermediate phases of myeloid differentiation. Importantly, since the levels of mRNA and protein expression do not necessarily coincide, we further demonstrated that RhoH protein levels also progressively decline. These results are consistent with HemaExplorer and DMAP *BloodSpot* data showing that during normal hematopoiesis *RHOH* expression progressively decreases as differentiation proceeds from common myeloid progenitors to granulocyte/monocyte progenitors and then to CD14+ monocytes [59, 60].

Analogous to what has been shown during lymphocyte activation, the repression of *RHOH* during myeloid differentiation along the monocytic pathway would be expected to release signalling through the Rho family and positively affect processes such as adhesion, chemotaxis, and *trans*‐endothelial migration critical to the inflammatory response [2, 4, 6, 8‐10, 12]. During the resolution of inflammation, monocytes that have terminally differentiated into macrophages either undergo apoptosis or become quiescent and take up residence in the tissues they infiltrate. Thus this terminal phase of monocytic differentiation is characterized by repression of inflammatory effector proteins such as the β2 integrin CD11b and CD93 [54, 55]. We demonstrate here that as monocytes terminally differentiate into macrophages *RHOH* expression is induced and that this induction causes a down‐regulation in the levels of both CD93 and CD11b protein. Indeed, transcriptome analysis indicates that *RHOH* induction regulates the expression of at least 42 genes known to affect functions characteristic of mature myeloid cells (Table 1). Taken together, our results indicate that myeloid differentiation along the monocytic pathway is driven by the bimodal expression of *RHOH*. A likely cause of this bimodal pattern is the reciprocal bimodal expression of the *RHOH* repressor *JUND* (Figure 4A, B) (GEO Series accession numbers GSE53691 & GSE5099) [14, 56, 57].

A molecular consequence of *RHOH* induction during the terminal phase of monocytic differentiation appears to be repression of the interlinked Cdc42 and Wnt intracellular signalling pathways (Figure 5). This finding in myeloid cells is consistent with previous reports in T‐lymphocytes showing *RHOH* inhibits Cdc42 signalling and its association with Wnt signalling through interaction with Kaiso and p120 catenin [18, 61, 62].

**FIGURE 7:**
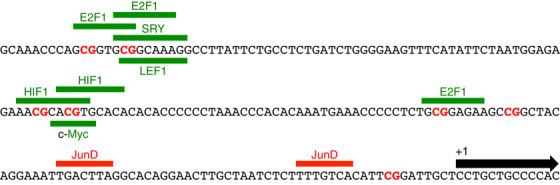
Nucleotide sequence of the P3 promoter of the human *RHOH* gene. The site of transcription initiated by the promoter is indicated as +1 with a black horizontal arrow depicting the 5′ end of exon 4 of the *RHOH* gene [14]. The location of potential binding sites for the transcription factor proteins c‐Myc, E2F1, HIF1, LEF1 and SRY that could activate the promoter are indicated with green horizontal bars and text [71, 72]. Each of these transcription factors is regulated by Wnt and/or Cdc42 signalling (Supplemental References 3). The location of binding sites for the JunD protein that represses the promoter are indicated with red horizontal bars and text [14]. The 7 CpG dinucleotides that could be methylated in AML are depicted in bold red text [70]

The *RHOH* gene can be transcribed from 3 distinct promoters, P1, P2, and P3 [14]. Where relative promoter strength has been compared, the P3 promoter has been shown predominant [14]. Analysis of the nucleotide sequence of this promoter identifies potential binding sites for HIF1, c‐Myc, SRY, E2F1, and LEF1 within the first 200 bp upstream of the transcription start site (Figure 7). All of these transcription factors have been shown to be regulated by Wnt and/or Cdc42 signalling (Supplemental References 3). Indeed, in the case of Wnt signalling, LEF1 plays a central role. Previously we have demonstrated that the P3 promoter can be repressed by the binding of the transcription factor protein JunD at two sites (Figure 7) [14]. Consequently, a molecular scenario can be envisioned in which *JUND* induction affects *RHOH* repression during the early and intermediate stages of myeloid differentiation leading to activation of Wnt signalling that in turn induces *RHOH* during the later differentiation stages through activation of factors such as LEF1 and supported by *JUND* repression.

Low expression of *RHOH* is an independent predictor of poor outcomes in AML [38]. Our differential microarray analysis indicates that this low expression likely drives pathogenesis by allowing the Cdc42 and Wnt pathways to be constitutively active (Figure 5). This contention is supported by aberrant signalling through both pathways being functionally linked to AML [61, 62]. In addition, our microarray data indicate that low *RHOH* expression results in high expression of the long non‐coding RNA *BASP1‐AS1* (lnc‐MYO10‐1) and low expression of the long non‐coding RNA *SNHG5* (GEO Accession number GSE138479). These expression patterns could cause *BASP1* repression through increased sponging of *BASP1* mRNA by *BASP1‐AS1* and increased expression of *microRNA 23a, 154, 182*, and *377* through decreased sponging by *SNHG5*. Repression of *BASP1* and induction of each of these microRNAs have been linked to the pathogenesis of AML [63, 64, 65, 66, 67, 68, 69].

A possible explanation of why *RHOH* is under‐expressed in AML comes from analysis of its P3 promoter and the finding that AML is characterized by the silencing of the promoters of Wnt antagonist genes through methylation [70]. Within the *RHOH* P3 promoter there are seven CpG dinucleotides that could be subject to methylation. Six of these dinucleotides are located within the potential binding sites for HIF1, c‐Myc, SRY, E2F1, or LEF1 (Figure 7). Consequently, CpG methylation would prevent the potential binding of one or more of these factors, possibly resulting in the failure of *RHOH* to be induced and subsequently the failure of Cdc42 and Wnt signalling to be repressed.

The hallmark of AML is the block of myeloblasts at various stages of their differentiation program. The most successful form of AML treatment is that for the acute promyelocytic subtype because its expression of the PML‐RARα fusion protein makes it amenable to differentiation induced by all‐*trans* retinoic acid [58]. Reconstitution of *RHOH* expression in the AML cell line OCI‐AML3 drives differentiation as assessed by the down‐regulation of CD93 and CD11b proteins. Such a finding raised the possibility that *RHOH* reconstitution might represent a new form of AML differentiation therapy. That OCI‐AML3 reconstituted with *RHOH* produces fewer and smaller tumours in immunocompromised mice than when not *RHOH* reconstituted demonstrates this new therapeutic strategy holds promise.

## CONFLICT OF INTEREST

Carl S. Shelley is President and CEO of Leukemia Therapeutics, LLC that is developing novel treatments for AML based on targeting the molecular consequences of aberrant *RHOH* repression.

## Supporting information

Supporting Information

Supporting Information

Supporting Information
