# Supplementary material for: Bimodal expression of RHOH during myelomonocytic differentiation: Implications for the expansion of AML differentiation therapy
Source: EJHaem. 2021 Jan 20;2(2):196–210. doi: 10.1002/jha2.128 (PMC9175762; doi:10.1002/jha2.128)

Supplemental Figure 1

Morphology of M4 AML myeloblasts following PMA treatment. Myeloblasts from a patient diagnosed with M4 AML and exhibiting more than 90% of blasts in the circulation were prepared by Ficoll gradient centrifugation. These cells were then cultured for 48 hours in RPMI-1640 supplemented with 20% heat-inactivated foetal calf serum, 4mM L-glutamine and either (**A**) 5 ng/mL PMA or (**B**) the equivalent volume of the PMA vehicle DMSO. Cells were photographed using a Leica DMi1 inverted microscope and x 80 magnification lens.

PMA **DMSO**

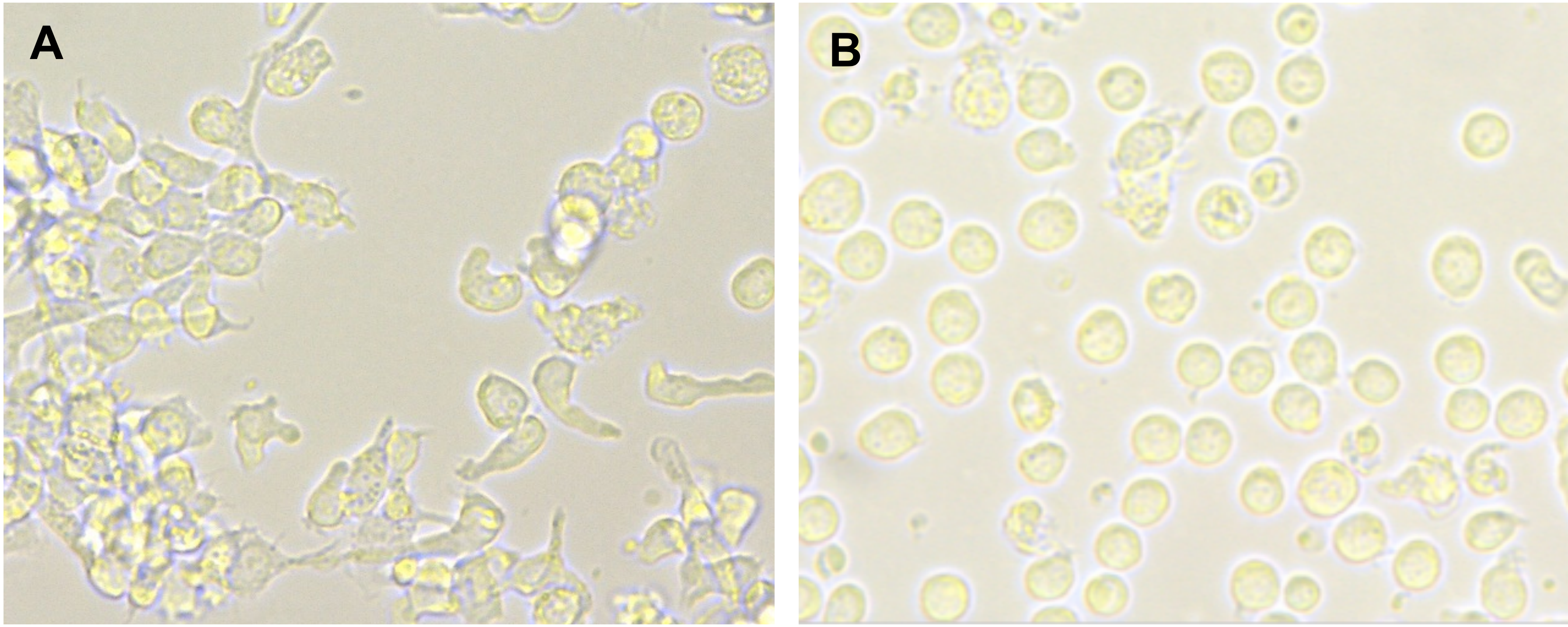

Supplement: Supplementary file 1 — Supporting Information [file JHA2-2-196-s003.pdf]
