# Supplementary material for: Bimodal expression of RHOH during myelomonocytic differentiation: Implications for the expansion of AML differentiation therapy
Source: EJHaem. 2021 Jan 20;2(2):196–210. doi: 10.1002/jha2.128 (PMC9175762; doi:10.1002/jha2.128)
Supplement: Supplementary file 2 — Supporting Information [file JHA2-2-196-s001.pdf]

Supplemental Table 1

Tumour volumes (mm^3) produced by subcutaneous injection of the cell lines OCI-Empty and OCI-RhoH into mice of the strain SHrN™ Hairless NOD.SCID

5 million cells injected

OCI-Empty		OCI-RhoH	
Mouse 1	163.2	Mouse 9	0
Mouse 2	156.8	Mouse 10	0
Mouse 3	0	Mouse 11	181.8
Mouse 4	279.4	Mouse 12	0
Mouse 5	133.7	Mouse 13	110.9
Mouse 6	216.6	Mouse 14	0
Mouse 7	74.5	Mouse 15	0
Mouse 8	145.8		

10 million cells injected

OCI-Empty		OCI-RhoH	
Mouse 16	1145.8*	Mouse 21	95.2
Mouse 17	68.4	Mouse 22	121.4
Mouse 18	156.5	Mouse 23	0
Mouse 19	163.1	Mouse 24	535.7*
Mouse 20	460.2	Mouse 25	0

Ordered Data Set: OCI-Empty
0, 68.4, 74.5, 133.7, 145.8, 156.5, 156.8, 163.1, 163.2, 216.6, 279.4, 460.2, 1145.8*

Ordered Data Set: OCI-RhoH
0, 0, 0, 0, 0, 0, 0, 95.2, 110.9, 121.4, 181.8, 535.7*

*Outlier values more than 1.5 times the interquartile range above the third quartile of their respective ordered data sets. These outlier values were not included in calculations of mean and standard error of the mean that are depicted in Figure 6.

There were no outlier values less than 1.5 times the interquartile range below the first quartile of the ordered data sets.
