# Supplementary material for: Bimodal expression of RHOH during myelomonocytic differentiation: Implications for the expansion of AML differentiation therapy
Source: EJHaem. 2021 Jan 20;2(2):196–210. doi: 10.1002/jha2.128 (PMC9175762; doi:10.1002/jha2.128)
Supplement: Supplementary file 3 — Supporting Information [file JHA2-2-196-s002.pdf]

Supplemental References 1

Table 1: Genes controlled by *RHOH* that regulate functions characteristic of mature myeloid cells

Consistent with the colour scheme of Table 1, genes down-regulated by *RHOH* are indicated by green text and genes up-regulated by *RHOH* are indicated by red text. Peer-reviewed publications have established a link between abnormal expression of each of these genes and the pathogenesis of AML.

ARNTL

Puram, R.V., Kowalczyk, M.S., de Boer, C.G., Schneider, R.K., Miller, P.G., McConkey, M., Tothova, Z., Tejero, H., Heckl, D., Järås, M., Chen, M.C., Li, H., Tamayo, A., Cowley, G.S., Rozenblatt-Rosen, O., Al-Shahrour, F., Regev, A. & Ebert, B.L. (2016) Core circadian clock genes regulate leukemia stem cells in AML. *Cell,* **165,** 303-316.

CD93

Iwasaki, M., Liedtke, M., Gentles, A.J. & Cleary, M.L. (2015) CD93 marks a non-quiescent human leukemia stem cell population and is required for development of MLL-rearranged acute myeloid leukemia. *Cell Stem Cell*, **17,** 412-421.

PRAM1

Lv, N., Qian, K., Liu, J., Wang, L.L., Li, Y.H. & Yu, L. (2018) Investigation of PRAM1 expression features and their clinical significance in AML via gene expression microarray database. *Zhongguo Shi Yan Xue Ye Xue Za Zhi,* **26,** 368-374.

PKC ε

Gobbi, G., Mirandola, P., Carubbi, C., Micheloni, C., Malinverno, C., Lunghi, P., Bonati, A. & Vitale, M. (2009) Phorbol ester-induced PKCepsilon down-modulation sensitizes AML cells to TRAIL-induced apoptosis and cell differentiation. *Blood,* **113,** 3080-3087.

Di Marcantonio, D., Martinez, E., Sidoli, S., Vadaketh, J., Nieborowska-Skorska, M., Gupta, A., Meadows, J.M., Ferraro, F., Masselli, E., Challen, G.A., Milsom, M.D., Scholl, C., Fröhling, S., Balachandran, S., Skorski, T., Garcia, B.A., Mirandola, P., Gobbi, G., Garzon, R., Vitale, M. & Sykes, S.M. (2018) Protein kinase C epsilon is a key regulator of mitochondrial redox homeostasis in acute myeloid leukemia. *Clinical Cancer Research*, **24,** 608-618.

PKC ζ

de Thonel, A., Bettaïeb, A., Jean, C., Laurent, G. & Quillet-Mary, A. (2001) Role of protein kinase C zeta isoform in Fas resistance of immature myeloid KG1a leukemic cells. *Blood*, **98,** 3770-3777.

TESC

Man, C.H., Lam, S.S., Sun, M.K., Chow, H.C., Gill, H., Kwong, Y.L. & Leung, A.Y. (2014) A novel tescalcin-sodium/hydrogen exchange axis underlying sorafenib resistance in FLT3-ITD+ AML. *Blood*, **123,** 2530-2539.

CDKN2C
Tschan, M.P., Peters, U.R., Cajot, J.F., Betticher, D.C., Fey, M.F. & Tobler, A. (1999) The cyclin-dependent kinase inhibitors p18INK4c and p19INK4d are highly expressed in CD34+ progenitor and acute myeloid leukaemic cells but not in normal differentiated myeloid cells. *British Journal of Haematology*, **106,** 644-651.

Schwaller, J., Pabst, T., Koeffler, H.P., Niklaus, G., Loetscher, P., Fey, M.F. & Tobler, A. (1997) Expression and regulation of G1 cell-cycle inhibitors (p16INK4A, p15INK4B, p18INK4C, p19INK4D) in human acute myeloid leukemia and normal myeloid cells. *Leukemia*, **11,** 54-63.

DUSP5P1
Zhou, L.Y., Yin, J.Y., Tang, Q., Zhai, L.L., Zhang, T.J., Wang, Y.X., Yang, D.Q., Qian, J., Lin, J. & Deng, Z.Q. (2015) High expression of dual-specificity phosphatase 5 pseudogene 1 (DUSP5P1) is associated with poor prognosis in acute myeloid leukemia. *International Journal of Clinical and Experimental Pathology*, **8,** 16073-16080.

FAT1
Garg, M., Nagata, Y., Kanojia, D., Mayakonda, A., Yoshida, K., Haridas Keloth, S., Zang, Z.J., Okuno, Y., Shiraishi, Y., Chiba, K., Tanaka, H., Miyano, S., Ding, L.W., Alpermann, T., Sun, Q.Y., Lin, D.C., Chien, W., Madan, V., Liu, L.Z., Tan, K.T., Sampath, A., Venkatesan, S., Inokuchi, K., Wakita, S., Yamaguchi, H., Chng, W.J., Kham, S.K., Yeoh, A.E., Sanada, M., Schiller, J., Kreuzer, K.A., Kornblau, S.M., Kantarjian, H.M., Haferlach, T., Lill, M., Kuo, M.C., Shih, L.Y., Blau, I.W., Blau, O., Yang, H., Ogawa, S. & Koeffler, H.P. (2015) Profiling of somatic mutations in acute myeloid leukemia with FLT3-ITD at diagnosis and relapse. *Blood*, **126,** 2491-2501.

NKD2
Li, X.X., Zhou, J.D., Zhang, T.J., Yang, L., Wen, X.M., Ma, J.C., Yang, J., Zhang, Z.H., Lin, J. & Qian, J. (2017) Epigenetic dysregulation of *NKD2* is a valuable predictor assessing treatment outcome in acute myeloid leukemia. *Journal of Cancer*, **8,** 460-468.

RPS3a
Hu, Z.B., Minden, M.D., McCulloch, E.A. & Stahl, J. (2000) Regulation of drug sensitivity by ribosomal protein S3a. *Blood,* **95,** 1047-1055.

SNHG5
Li, J. & Sun, C.K. (2018) Long noncoding RNA SNHG5 is up-regulated and serves as a potential prognostic biomarker in acute myeloid leukemia. *European Review for Medical and Pharmacological Sciences*, **22,** 3342-3347.

Wang, D., Zeng, T., Lin, Z., Yan, L., Wang, F., Tang, L., Wang, L., Tang, D., Chen, P. & Yang, M. (2020) Long non-coding RNA SNHG5 regulates chemotherapy resistance through the miR-32/DNAJB9 axis in acute myeloid leukemia. *Biomédecine & Pharmacothérapie*, **123,** 109802.

Supplemental References 2

Figure 5: Regulators of Cdc42 and Wnt signalling controlled by *RHOH*
Particular pairs of proteins that peer-reviewed publications have linked in Cdc42 and/or Wnt signalling are indicated by coloured text. Consistent with the colour scheme of Figure 5, core components of Cdc42 and Wnt signalling are indicated by blue text, agonists down-regulated by *RHOH* are indicated by green text and antagonists up-regulated by *RHOH* are indicated by red text.

Dvl / PKC ζ
Velázquez, D.M., Castañeda-Patlán, M.C. & Robles-Flores, M. (2017) Dishevelled stability is positively regulated by PKCζ-mediated phosphorylation induced by Wnt agonists. *Cellular Signalling*, **35**,107-117.

Par6 / PKC ζ
Noda, Y., Takeya, R., Ohno, S., Naito, S., Ito, T. & Sumimoto, H. (2001) Human homologues of the Caenorhabditis elegans cell polarity protein PAR6 as an adaptor that links the small GTPases Rac and Cdc42 to atypical protein kinase C. *Genes to Cells*, **6**,107-119.

Kim, S.K. (2000) Cell polarity: new PARtners for Cdc42 and Rac. *Nature Cell Biology*, **2**, E143-E145.

Qiu, R.G., Abo, A. & Martin, G.S. (2000) A human homolog of the C. elegans polarity determinant Par-6 links Rac and Cdc42 to PKCzeta signaling and cell transformation. *Current Biology*,**10**, 697-707.

Lin, D., Edwards, A.S., Fawcett, J.P., Mbamalu, G., Scott, J.D. & Pawson, T. A. (2000) Mammalian PAR-3-PAR-6 complex implicated in Cdc42/Rac1 and αPKC signalling and cell polarity. *Nature Cell Biology*, **2**, 540-547.

Joberty, G., Petersen, C., Gao, L. & Macara, I.G. (2000) The cell-polarity protein Par6 links Par3 and atypical protein kinase C to Cdc42. *Nature Cell Biology*, **2**, 531-539.

Borg 5 / PKC ζ
Farrugia, A.J. & Calvo, F. (2016) The Borg family of Cdc42 effector proteins Cdc42EP1-5. *Biochemical Society Transactions*, **44**, 1709-1716.

CDC42 / Borg 5 & Borg 2
Joberty, G., Perlungher, R.R. & Macara, I.G. (1999) The Borgs, a new family of Cdc42 and TC10 GTPase-interacting proteins. *Molecular and Cellular Biology*, **19**, 6585-6597.

Cohen, S., Kovari, D.T., Wei, W., Keate, R., Curtis, J.E. & Nie, S. (2018) Cdc42 regulates the cellular localization of Cdc42ep1 in controlling neural crest cell migration. *Journal of Molecular and Cellular Biology*, **10**, 376-387.

Farrugia, A.J. & Calvo, F. (2017) Cdc42 regulates Cdc42EP3 function in cancer-associated fibroblasts. *Small GTPases*, **8**, 49-57.

Septin 7 / Borg 5 & Borg 2
Farrugia, A.J. & Calvo, F. (2016) The Borg family of Cdc42 effector proteins Cdc42EP1-5. *Biochemical Society Transactions*, **44**, 1709-1716.

Septin 7 / Septin 11
Nagata, K., Asano, T., Nozawa, Y. & Inagaki, M. (2004) Biochemical and cell biological analyses of a mammalian septin complex, Sept7/9b/11. *Journal of Biological Chemistry*, **279**, 55895-55904.

c-Met / Septin 11
Mostowy, S., Janel, S., Forestier, C., Roduit, C., Kasas, S., Pizarro-Cerdá, J., Cossart, P. & Lafont, F. (2011) A role for septins in the interaction between the Listeria monocytogenes invasion protein InlB and the Met receptor. *Biophysical Journal*, **100**, 1949-1959.

CDC42 / PICK1
Rocca, D.L. & Hanley, J.G. (2015) PICK1 links AMPA receptor stimulation to Cdc42. *Neuroscience Letters*, **585**,155-159.

Dvl / NKD2
Hu, T., Li, C., Cao, Z., Van Raay, T.J., Smith, J.G., Willert, K., Solnica-Krezel, L. & Coffey, R.J. (2010) Myristoylated Naked2 antagonizes Wnt-beta-catenin activity by degrading Dishevelled-1 at the plasma membrane. *Journal of Biological Chemistry*, **285**,13561-13568.

Dvl & β-Catenin / RMRP
Sun, X., Zhang, R., Liu, M., Chen, H., Chen, L., Luo, F., Zhang, D., Huang, J., Li, F., Ni, Z., Qi, H., Su, N., Jin, M., Yang, J., Tan, Q., Du, X., Chen, B., Huang, H., Chen, S., Yin, L., Xu, X., Deng, C., Luo, L., Xie, Y. & Chen, L. (2019) Rmrp mutation disrupts chondrogenesis and bone ossification in zebrafish model of cartilage-hair hypoplasia via enhanced Wnt/β-catenin signaling. *Journal of Bone and Mineral Research*, **34**, 2101-2116.

Dvl & β-Catenin / BMAL1
Guo, B., Chatterjee, S., Li, L., Kim, J.M., Lee, J., Yechoor, V.K., Minze, L.J., Hsueh, W. & Ma, K. (2012) The clock gene, brain and muscle Arnt-like 1, regulates adipogenesis via Wnt signaling pathway. *FASEB Journal*, **26**, 3453-3463.

β-Catenin / TBL1X & TBL1Y
Dimitrova, Y.N., Li, J., Lee, Y.T., Rios-Esteves, J., Friedman, D.B., Choi, H-J., Weis, W.I., Wang, C-H. & Chazin, W.J. (2010) Direct ubiquitination of beta-catenin by Siah-1 and regulation by the exchange factor TBL1. *Journal of Biological Chemistry*, **285**, 13507-13516.

Li, J. & Wang, C.Y. (2008) TBL1-TBLR1 and beta-catenin recruit each other to Wnt target-gene promoter for transcription activation and oncogenesis. *Nature Cell Biology*, **10**, 160-169.

Choi, H.K., Choi, K.C., Yoo J.Y., Song, M., Ko, S.J., Kim, C.H., Ahn, J-H., Chun, K-H., Yook, J.I. & Yoon, H-G. (2011) Reversible SUMOylation of TBL1-TBLR1 regulates β-catenin-mediated Wnt signaling. *Molecular Cell*, **43**, 203-216.

β-Catenin / FAT1
Morris, L.G., Kaufman, A.M., Gong, Y., Ramaswami, D., Walsh, L.A., Turcan, S., Eng, S., Kannan, K., Zou, Y., Peng, L., Banuchi, V.E., Paty, P., Zeng, Z., Vakiani, E., Solit, D., Singh, B., Ganly, I., Liau, L., Cloughesy, T.C., Mischel, P.S., Mellinghoff, I.K. & Chan, T.A. (2013) Recurrent somatic mutation of FAT1 in multiple human cancers leads to aberrant Wnt activation. *Nature Genetics*, **45**, 253-261.

Dvl / FOXK1
Wang, W., Li, X., Lee, M., Jun, S., Aziz, K.E., Feng, L., Tran, M.K., Li, N., McCrea, P.D., Park, J-Il. & Chen, J. (2015) FOXKs promote Wnt/β-catenin signaling by translocating DVL into the nucleus. *Developmental Cell*, **32**, 707-718.

Ji, Z.G., Jiang, H.T. & Zhang, P.S. (2018) FOXK1 promotes cell growth through activating wnt/β-catenin pathway and emerges as a novel target of miR-137 in glioma. *American Journal of Translational Research*, **10**, 1784-1792.

FOXK1 / SRF
Freddie, C.T., Ji, Z., Marais, A. & Sharrocks, A.D. (2007) Functional interactions between the Forkhead transcription factor FOXK1 and the MADS-box protein SRF. *Nucleic Acids Research*, **35**, 5203-5212.

β-Catenin / SRF
Choi, H.N., Kim, K.R., Lee, J.H., Park, H.S., Jang, K.Y., Chung, M.J., Hwang, S.E., Yu, H.C. & Moon, W.S. (2009) Serum response factor enhances liver metastasis of colorectal carcinoma via alteration of the E-cadherin/beta-catenin complex. *Oncology Reports*, **21**, 57-63.

CDC42 / SRF
Hill, C.S., Wynne, J. & Treisman, R. (1995) The Rho family GTPases RhoA, Rac1, and CDC42Hs regulate transcriptional activation by SRF. *Cell*, **81**, 1159-1170.

Reymond, N., Im, J.H., Garg, R., Vega, F.M., Borda d'Agua, B., Riou, P., Cox, S., Valderrama, F., Muschel, R.J. & Ridley, A.J. (2012) Cdc42 promotes transendothelial migration of cancer cells through β1 integrin. *Journal of Cell Biology*, **199**, 653-668.

Hao, S., Kurosaki, T. & August, A. (2003) Differential regulation of NFAT and SRF by the B cell receptor via a PLCgamma-Ca(2+)-dependent pathway. *EMBO Journal*, **22**, 4166-4177.

PKC ε / SRF
Soh, J.W., Lee, E.H., Prywes, R. & Weinstein, I.B. (1999) Novel roles of specific isoforms of protein kinase C in activation of the c-fos serum response element. *Molecular and Cellular Biology*, **19**, 1313-1324.

β-Catenin / PKC ε
Duong, M., Yu, X., Teng, B., Schroder, P., Haller, H., Eschenburg, S. & Schiffer, M. (2017) Protein kinase C ϵ stabilizes β-catenin and regulates its subcellular localization in podocytes. *Journal of Biological Chemistry*, **292**, 12100-12110.

IQGAP1 / PKC ε

Grohmanova, K., Schlaepfer, D., Hess, D., Gutierrez, P., Beck, M. & Kroschewski, R. (2004) Phosphorylation of IQGAP1 modulates its binding to Cdc42, revealing a new type of rho-GTPase regulator. *Journal of Biological Chemistry*, **279**, 48495-48504.

Supplemental References 3

Figure 7: Nucleotide sequence of the P3 promoter of the human *RHOH* gene
Potential binding sites for the transcription factor proteins c-Myc, E2F1, HIF1, LEF1 and SRY were identified in the P3 promoter of the human *RHOH* gene using the computer program PROMO (Messeguer *et al*, 2002; Farré *et al*, 2003). Peer-reviewed publications demonstrate that each of these transcription factors is regulated by Wnt and/or Cdc42 signalling.

c-Myc
He, T.C., Sparks, A.B., Rago, C., Hermeking, H., Zawel, L., da Costa, L.T., Morin, P.J., Vogelstein, B. & Kinzler, K.W. (1998) Identification of c-MYC as a target of the APC pathway. *Science*, **281,** 1509-1512.

E2F1
Yu, S., Yerges-Armstrong, L.M., Chu, Y., Zmuda, J.M. & Zhang, Y. (2013) E2F1 effects on osteoblast differentiation and mineralization are mediated through up-regulation of frizzled-1. *Bone*, **56,** 234-241.

Abramova, M.V., Zatulovskiy, E.A., Svetlikova, S.B., Kukushkin, A.N. & Pospelov, V.A. (2010) e2f1 gene is a new member of Wnt/beta-catenin/Tcf-regulated genes. *Biochemical and Biophysical Research Communications*, **391,** 142-146.

Zhou, F., Zhang, L., Gong, K., Lu, G., Sheng, B., Wang, A., Zhao, N., Zhang, X. & Gong, Y. (2008) LEF-1 activates the transcription of E2F1. *Biochemical and Biophysical Research Communications*, **365,** 149-153.

HIF1
Xue, Y., Bi, F., Zhang, X., Zhang, S., Pan, Y., Liu, N., Shi, Y., Yao, X., Zheng, Y. & Fan, D. (2006) Role of Rac1 and Cdc42 in hypoxia induced p53 and von Hippel-Lindau suppression and HIF1alpha activation. *International Journal of Cancer*, **118,** 2965-2972.

LEF1
Santiago, L., Daniels, G., Wang, D., Deng, F.M. & Lee, P. (2017) Wnt signaling pathway protein LEF1 in cancer, as a biomarker for prognosis and a target for treatment. *American Journal of Cancer Research*, **7,** 1389-1406.

Clevers, H. & van de Wetering, M. (1997) TCF/LEF factor earn their wings. *Trends in Genetics*, **13,** 485-489.

MacDonald, B.T., Tamai, K. & He, X. (2009) Wnt/beta-catenin signaling: components, mechanisms, and diseases. *Developmental Cell*, **17,** 9-26.

Filali, M., Cheng, N., Abbott, D., Leontiev, V. & Engelhardt, J.F. (2002) Wnt-3A/beta-catenin signaling induces transcription from the LEF-1 promoter. *Journal of Biological Chemistry*, **277,** 33398-33410.

Hovanes, K., Li, T.W., Munguia, J.E., Truong, T., Milovanovic, T., Marsh, J.L., Holcombe, R.F. & Waterman, M.L. (2001) Beta-catenin-sensitive isoforms of lymphoid enhancer factor-1 are selectively expressed in colon cancer. *Nature Genetics*, **28,** 53-57.

SRY
Jordan, B.K., Mohammed, M., Ching, S.T., Délot, E., Chen, X.N., Dewing, P., Swain, A., Rao, P.N., Elejalde, B.R. & Vilain, E. (2001) Up-regulation of WNT-4 signaling and dosage-sensitive sex reversal in humans. *American Journal of Human Genetics*, **68,** 1102-1109.

Mizusaki, H., Kawabe, K., Mukai, T., Ariyoshi, E., Kasahara, M., Yoshioka, H., Swain, A. & Morohashi, K. (2003) Dax-1 (dosage-sensitive sex reversal-adrenal hypoplasia congenita critical region on the X chromosome, gene 1) gene transcription is regulated by wnt4 in the female developing gonad. *Molecular Endocrinology*, **17,** 507-519.
